# Evaluating Social Assistive Robots in Clinical Nursing Care: Mixed Method Pilot Study on Health Care Workers’ Perceptions and Adoption

**DOI:** 10.2196/70305

**Published:** 2025-08-01

**Authors:** Janika Leoste, Kadi Lubi, Kristel Marmor, Katrin Kangur

**Affiliations:** 1 IT College, Tallinn University of Technology, Ehitajate tee 5, Tallinn, 19086, Estonia, 372 5045081; 2 School of Educational Sciences, Tallinn University, Tallinn, Estonia; 3 Department of Healt Technologies, Tallinn University of Technology, Tallinn, Estonia

**Keywords:** aging population, nursing, socially assistive robots, technology acceptance in healthcare, human-robot interaction

## Abstract

**Background:**

The growing demand for older adults care due to aging populations and health care workforce shortages requires innovative solutions. Socially assistive robots (SARs) are increasingly explored for their potential to reduce workload by handling routine tasks. Yet, adoption can be hindered by various health care workers’ concerns.

**Objective:**

This study examined the perceptions of health care workers toward SARs before and after a pilot use in a clinical nursing care setting. The study focused on SAR usability, emotional appropriateness, and readiness for adoption.

**Methods:**

A mixed methods pilot study was conducted at the East Tallinn Central Hospital’s Nursing Care Clinic in collaboration with Tallinn University of Technology. The TEMI v3 (Robotemi) robot was used for 2 weeks for visitor guidance, goods delivery, and patrolling tasks. Health care workers filled in pre- and postintervention questionnaires with Likert-scale items and a broad open-ended question. Quantitative data were analyzed for changes in perceived safety, trust, and usability. Qualitative data underwent thematic analysis to understand participants’ opinions.

**Results:**

Out of 45 involved health care workers, 20 completed the pretest questionnaire, and 5 completed the posttest questionnaire (a 75% attrition). Pretest results show that 17 of 20 (85%) participants had limited previous exposure to SARs and mixed perceptions of their role, with 9 (45%) viewing SARs as machines and 6 (30%) as somewhat human-like. Although 60% believed SARs could become mainstream within 5‐10 years, there were concerns about the robot’s emotional adequacy and job displacement. Posttest findings showed increased confidence in SARs, with all respondents perceiving them as safe tools. Qualitative results indicate improved trust and readiness to integrate SARs into daily routines, with 4 out of 5 (80%) being willing to advocate for SAR use. Still, participants noted limited impact on facilitating their jobs.

**Conclusions:**

The study indicates that short-term collaboration with SARs can enhance health care workers’ confidence and their readiness for adoption. However, actual use would need proper emotional adequacy from the robot and aligning its functionalities with specific care needs. The future studies need to examine long-term impacts on care quality and job satisfaction, and also strategies to address generational differences and technophobia among health care staff. Transparent communication and proper training are required to ensure acceptance.

## Introduction

### Background

The growing integration of socially assistive robots (SARs) into older adults’ care settings offers transformative potential to address workforce resource limitations and improve care delivery [1]. Throughout Europe, including Estonia, the aging population has placed a significant burden on health care systems, the demand for older adults’ care is growing while the shortages of qualified labor are increasing, and existing staff experience burnout due to high workloads and emotional demands [2,3]. In this context, robot assistants such as the TEMI v3 (Robotemi) robot are emerging as viable solutions to assist health care workers in performing routine and physically demanding tasks, allowing workers to focus on more complex and interpersonal care responsibilities. SARs, with their wide range of designs (humanoid, animal-like, telepresence, etc), can provide assistance and improve patient engagement in care settings [4].

A typical SAR can navigate autonomously or semiautonomously, it can recognize voice and process natural language, and it can also be used for enhanced video conferencing with improved social presence, potentially improving thus communication and interaction in hospitals and homes [5,6]. For instance, the TEMI robot can autonomously navigate predefined areas, use telepresence to relay interaction between patients and medical personnel or their relatives, and perform simple service tasks [7]. SARs are designed to assist users through social interaction, focusing on cognitive and social assistance. These robots interact with humans using natural language, body language, and social behaviors, enhancing psychological and emotional well-being and complementing traditional caregiving services [1]. In health care, SARs provide services that directly benefit patients, health care professionals, and caregivers. They are particularly useful in supporting older adults and patients with chronic illnesses, who require continuous monitoring and social interaction. SAR functions include remote patient monitoring, facilitating swift responses to incidents like falls or medical emergencies, measuring vital signs (eg, pulse, blood pressure, and oxygen saturation), and assisting in remote consultations between patients and health care providers [8,9]. These robots are also used to combat social isolation, significantly enhancing patients’ sense of companionship and social presence in long-term care facilities or for individuals confined at home [10].

Leaning on the context of using SARs in health care to empower health care workers’ professional capabilities, the aim of this study was to examine the effects of using the TEMI v3 robot assistants for 2 weeks in a nursing clinic’s daily practices to examine the feasibility and usability of SARs as perceived by health care workers. The study is arranged as follows: (1) first, we open the Theoretical Background by examining studies about the real-world use of SARs in health care, discussing appropriate technology acceptance models and relevant concepts, and exploring the challenges in adopting novel technologies in health care. Then, in (2) the Methods, we discuss the study design, sample, and procedure, describe the methods for collecting and analyzing data, and present ethical considerations. Followed in (3) the Results, we give a detailed overview of our quantitative and qualitative results, together with a comparison of pre- and posttest findings. And we conclude with (4) the Discussion that includes a description of the study’s limitations and suggestions for future research and practice.

### Real-World Use of SARs in Health Care

Although SARs with their modern functionalities are relatively new technologies, there are already a few studies that have investigated their efficacy in health care. The results of these studies give knowledge of how SARs can enhance patient well-being, support health care professionals, and improve the overall quality of care.

The CARESSES (Culture-Aware Robots and Environmental Sensor Systems for Elderly Support) randomized controlled trial was a good example of such studies [11]. This trial explored the impact of culturally competent SARs (in this case, the Pepper robots) on older adults in care homes. The study involved 33 residents across facilities in England and Japan, who interacted with SARs that were programmed with varying levels of cultural competence. The findings indicated that participants who engaged with culturally competent robots exhibited significant improvements in emotional well-being, as measured by the SF-36 emotional well-being subscale, compared with those receiving standard care. While changes in loneliness scores were observed, they did not reach statistical significance. These results suggest that customizing robot interactions to consider the users’ cultural backgrounds can enhance the psychological benefits of SARs in older adult care settings [11].

In pediatric health care, SARs have been used to alleviate distress and anxiety related to medical procedures. A systematic review by Trost et al [12] examined the effectiveness of SAR interventions in reducing pain and emotional discomfort among children. The review concluded that while SARs show promise in reducing distress and anxiety, evidence regarding their impact on pain reduction remains inconclusive. The authors emphasized the need for further research to establish standardized protocols and assess the long-term benefits of SARs in pediatric settings.

For individuals with dementia, SARs have been used to evoke positive emotional responses and reduce apathy. A pilot study by Otaka et al [13] investigated the immediate emotional reactions of nursing home residents with dementia to multisensory stimuli presented by SARs. Using facial expression analysis, the study found that participants exhibited increased expressions of happiness, particularly when engaging with animal-like or doll-type robots that provided combined visual, auditory, and tactile stimuli. These findings highlight the potential of SARs to enhance emotional engagement in dementia care, especially when designed to offer multimodal interactions.

In the realm of rehabilitation, SARs have been integrated into poststroke therapy to provide personalized coaching and monitoring. Lee et al [14] developed an interactive SAR system that combines neural network and rule-based models to assess patients’ rehabilitation exercises in real time. The system was evaluated with 15 poststroke survivors, demonstrating its ability to adapt to individual patient needs and provide corrective feedback. Participants reported increased motivation and satisfaction with the robot-assisted therapy, suggesting that SARs can play a valuable role in enhancing rehabilitation outcomes.

These studies indicate that SARs can be versatile when addressing health care challenges. The robots can improve the quality of care and support in medical settings when their interactions are programmed to consider specific patient needs and cultural contexts. From the technical point of view, however, the introduction and acceptance of SARs in health care settings face several challenges. These include the necessity for robust and reliable internet connectivity, technical support for robot operation, and modifications in physical spaces to accommodate robot navigation. There are also limitations related to sensory input, such as restricted visual and auditory capabilities, potentially leading to communication challenges and reduced social presence compared with face-to-face interactions [1,15]. In addition, the introduction of SARs raises significant emotional and professional concerns for both health care professionals and patients. Next, we examine these issues while addressing specific fears associated with SARs such as job relocation, loss of human contact, emotional unsuitability to care settings, and a general inability to adapt to new technologies. These concepts frame the complexities of SAR integration, demonstrating both the opportunities and challenges of SARs in the health care sector.

### Technology Acceptance in Health Care

The adoption of SARs in health care can be studied through widely established technology acceptance models, mainly the Technology Acceptance Model (TAM) [16] and the Unified Theory of Technology Acceptance and Use (UTAUT) [17]. These models emphasize the importance of perceived usefulness and ease of use, suggesting that when users find new technologies useful and easy to understand, their willingness to adopt these technologies increases. Research also emphasizes that technology readiness, that is, user readiness and willingness to adopt and engage with new technology, is a critical factor in health care settings [18,19]. In health care, the application of technology such as SARs brings additional considerations beyond functionality and ease of use, particularly the socio-emotional implications of care [19,20]. Social exchange theories also provide a basis for understanding how the relational aspects of care affect technology acceptance, emphasizing that perceived coldness or impersonality of robots can hinder their acceptance in contexts that require empathy and warmth [21]. Health care workers often need special training and time to adapt to SAR technology, which is central to reducing fear and increasing confidence in robotic assistance. In addition, participatory training can significantly improve technology adoption and smooth transitions to new work processes [11]. Our pilot study, based on the use of the TEMI v3 robot, emphasized these readiness principles by adding predeployment training and scenario-based familiarization that allowed personnel to engage in SARs in a controlled environment before full deployment.

### Challenges in Adopting New Technologies

One of the main concerns regarding the implementation of SARs in health care is the fear of job displacement. Many health care workers perceive the introduction of robots not only as a complement to their roles, but as a potential threat to workplace safety. Such fears are supported by research showing that job displacement problems are common among workers in automated industries [22]. In the health care context, where relationships and personalized communication are critical, these concerns are even more acute [23]. Although SARs are designed to support tasks that do not require human contact, employees may feel uncertain about the boundaries of their roles as the capabilities of SARs expand. Thus, clear communication of SAR’s supportive, not substitute, role may help alleviate this fear.

Another barrier to integrating SARs into health care is the reluctance or inability of individual staff members to adapt to new technologies, especially those with limited digital skills or existing technophobia. Technophobia, or the fear of engaging with new technologies, can manifest in many ways, from general anxiety about using technology to concerns about one’s ability to learn and adapt to robotic systems [24]. This fear is especially pronounced among older health care workers or those who are limited by digital tools. Research shows that this lack of technological readiness can hinder the successful deployment of SARs, highlighting the need for robust training programs that build familiarity and confidence among staff [20,25].

Health care providers and patients express serious concerns about the perceived emotional inadequacy of SARs in sensitive treatment contexts. The “human touch” is a key aspect of geriatric care, as many older adult patients depend on interpersonal interactions for emotional support and social bonding. Research has shown that SARs are often perceived to lack the warmth and empathy essential to their caregiving roles, leading to resistance from both patients and caregivers [23]. Although SARs can perform tasks efficiently, their inability to mimic human empathy or intuition limits their suitability for roles involving complex emotional needs, such as comforting an anxious patient or providing personalized encouragement during rehabilitation exercises.

## Methods

### Study Design

This study used a quasi-experimental mixed methods design to assess health care workers’ perceptions and experiences with SARs, using the TEMI v3 robot as an example, within a real-world health care setting. Applying mixed methods in pilot feasibility studies is justified when integrating quantitative and qualitative data could provide a comprehensive understanding of implementation feasibility, acceptability, and practical implications of new interventions [26]. A quasi-experimental design was specifically chosen due to the study’s exploratory nature, the limited duration of 2 weeks, practical constraints in the clinical setting, and the intention to gather initial insights into changes in staff perceptions and experiences following robot deployment. The pilot study was part of a broader registered social scientific feasibility research (the ethics committee registration details are provided under “Ethical Considerations”), conducted at the East Tallinn Central Hospital’s Nursing Care Clinic in Estonia, in collaboration with Tallinn University of Technology. The design involved pre- and postintervention questionnaires, including both closed and open-ended questions, allowing for quantitative comparisons and qualitative insights into health care workers’ attitudes and readiness concerning SARs before and after exposure. In addition, scenario-based role assignments were used, simulating practical usage situations to capture nuanced staff reactions, emotional responses, and potential concerns in a controlled yet realistic environment. Over a period of 2 weeks in the summer of 2024, 3 distinct SAR usage scenarios (visitor guidance, delivery assistance, and facility patrolling) were trialed. These scenarios (see “Procedure” for more details) were carefully selected based on the robot’s technical abilities and preliminary discussions with health care staff and administrators, reflecting ancillary tasks that would support rather than replace the primary caregiving roles of human staff.

Our study addressed the challenges described in the “Challenges in Adopting New Technologies” section in the following manner. First, we strategically deployed SARs in ancillary rather than primary health care roles; for example, the robots were used for visitor guidance, delivery, and patrolling functions. These tasks complement people’s care by reducing workload without compromising the interpersonal aspects that are central to caring for older adults. During our pilot, health care administrators played an essential role in clearly communicating to staff members the specific purpose and scope of the SARs deployment. Administrators were involved because their responsibility in decision-making processes and in organizational change management put them in the position where they could address the personnel’s concerns about job security and explain how SARs are used strictly as supportive tools instead of being replacements for human staff members. To avoid any perceived power imbalance, administrators took part in structured meetings, where dialogue and feedback regarding the roles assigned to SARs were encouraged. These discussions were helpful for keeping transparency and supporting collaboration where employees felt safe to present their suggestions and concerns. Next, the robot’s tasks were structured to assist rather than replace human interaction. For example, a robot’s role as a tour guide or logistical assistant fulfills functional needs without infringing on the emotionally charged tasks performed by health care providers. This design choice reflects a “complementarity model” where SARs are positioned to enhance caregivers’ capabilities without diminishing the importance of human connection [27]. Finally, we implemented short training sessions, allowing staff to interact with the robot in a supportive, low-stakes environment before integrating SARs into daily operations, in order to increase user confidence, reduce technophobia, and promote an inclusive environment that accommodates different levels of digital competence. In addition, involving employees directly in defining the roles and tasks for SARs has been shown to be effective in reducing resistance, as it empowers them to shape how the technology fits into their existing workflows, rather than feeling like it is being imposed by administrative decision-making alone [11].

### Participants

The sample consisted of 45 employees of East Tallinn Central Hospital’s long-term Nursing Care Clinic, all of whom were invited to participate in the study voluntarily. A total of 20 of them completed the pretest and 5 completed the posttest questionnaire. The participants represented a variety of roles in the clinic, including nurses, care workers, and other health care professionals, and administrative staff who interact with patients and the hospital environment on a daily basis. To ensure inclusiveness, all employees who could encounter the TEMI v3 robot in their daily activities were given the opportunity to participate. Anonymity was maintained throughout the study to encourage honest feedback. To this end, throughout the study, anonymity of responses to the questionnaires was emphasized, and for this purpose, gender information was excluded as the employees at this clinic were predominantly female. Demographic information, including age range but excluding gender, was collected to understand differences in employee technology adaptation.

### Procedure

#### Preintervention Training and Questionnaire

Before SAR implementation, participants underwent short training to familiarize themselves with the functions, controls, and user interface of the TEMI v3 robot. The training covered robot navigation, object transport, patrol functionality, and interaction options. After the training, participants completed a preintervention questionnaire to capture initial attitudes, perceived ease of use, usefulness, and potential fears (eg, job relocation and loss of human contact in caregiving). This questionnaire included both quantitative items on a modified Likert scale and an open-ended question for qualitative feedback.

#### Application of the Robot in 3 Scenarios

During a 2-week period, TEMI v3 was used in the clinic to perform 3 specific functions, each tailored to complement routine tasks without replacing human care:

- Visitor guidance (Scenario 1): TEMI v3 was programmed to navigate visitors to specific locations in the clinic (eg, patient rooms and nurse stations) on request. Visitors could select destinations on the robot’s interface screen, after which TEMI guided them to the desired location. This task required minimal interpersonal interaction on the part of the staff while allowing feedback on SAR effectiveness and usability to be monitored.
- Goods delivery (scenario 2): TEMI v3 was equipped with a tray for the delivery of light items such as personal items or documents between rooms or departments. Workers placed items on TEMI’s tray, selected a delivery location through the robot’s interface, and TEMI delivered the items autonomously. This scenario simulated the use of SARs for logistical support in day-to-day maintenance.
- Patrol functionality (scenario 3): TEMI v3 performed patrol duties in the corridors of the clinic during the evening and night shifts, moving between predetermined points. The patrol function allowed staff to monitor corridors via TEMI’s real-time video feed without compromising patient privacy as no data were recorded. This feature provided additional security and oversight without directly replacing employee roles.

#### Postintervention Questionnaire and Feedback Collection

At the end of the 2-week period, a follow-up seminar was held to allow participants to discuss their experiences with TEMI v3 in a group. This provided additional insight into employee concerns, perceived benefits, and the emotional and practical impact of SARs on their work environment. During the seminar, the participants also completed a postintervention questionnaire that had the same questions as the preintervention questionnaire (including the open-ended question for qualitative data), recording changes in participants’ perceptions of SAR use, perceived usefulness, ease of use, and emotional impact. As participation in the study was voluntary, the postintervention questionnaires were made available to the participants who were present at the seminar, and to those who wanted to add their reflections, although they were not able to be present. The pre- and postintervention qualitative data were used together to extract as many potential themes to describe the perceived roles of SARs or their influence on involved people (we did not measure changes in these perceptions).

### Measures and Data Analysis

#### Pre- and Postintervention Questionnaires, Quantitative Data

The pre- and posttest questionnaires that were filled in by the participants according to the procedure described in the previous subsection used Likert-scale items (n=19) to measure participants’ perceptions toward SAR adoption, perceived usefulness, perceived ease of use, and perceived threats (eg, fear of job transfer, emotional inadequacy, and inability to adapt). The prequestionnaire established the basis for these perceptions, while the postquestionnaire aimed to assess changes in perception after the 2-week intervention. The questionnaires included both quantitative items for analysis and an open-ended question for qualitative exploration of nuanced responses and issues. The questionnaires were based on established frameworks and approaches to understand user perceptions and readiness to adopt SAR in health care. The TAM and the UTAUT were the underlying models. TAM emphasizes the importance of perceived usefulness and ease of use in technology acceptance [16], while UTAUT extends this to include social influence and facilitating conditions as key determinants of user intentions and behavior [17]. In addition, elements from human-robot interaction (HRI) and social presence theory were added to assess participants’ perceptions of the SAR’s “personality” and the social dynamics of their interactions with it. This is critical in health care, where the perceived warmth and empathy of technology can influence acceptance, particularly in aged care settings [21]. Questions assessing trust, security, and reliability were based on the principles of trust in automation, which consider predictability and reliability important for developing user trust in automated systems [28,29]. Finally, the concept of technology readiness was integrated to measure health professionals’ confidence and propensity to engage in SARs in line with the Technology Readiness Index (TRI), which measures a person’s readiness to adopt new technologies [30] (Textbox 1).

Textbox 1. **Metrics captured by the questionnaire.**

Specifically, the questionnaires included:

• Demographic information (3 items): length of employment in health care (categories from less than 1 year to more than 5 years); age group (up to 30 years, 31-50 years, and 51 years and older); frequency of previous interactions with robots in education or work contexts; and frequency of previous interactions with robots in education or work contexts.

• Perceptions of future use of robots (2 items): expectations of robot usage in health care within 5-10 years; Perceptions of robots’ roles as colleagues, competitors, or tools.

• Interaction experience with robot during trial (3 items): frequency of interactions with the robot during the trial period; perception of the robot as a machine or human-like during interactions; and general evaluation of robot usage frequency.

• Robot interaction quality and usability (7 items): ease of cooperation with the robot; clarity and comprehensibility of communication with the robot; suitability of robot’s physical position and height; perceived safety while interacting with the robot; trustworthiness of the robot; pleasantness of interaction; and confidence while interacting with the robot.

• Readiness and acceptance of robot integration (4 items): willingness to use robots regularly in daily work; perceived potential of robots to simplify work tasks; willingness to actively support the introduction of robots into the workplace; and expectation of robots’ long-term impact on work.

The collected paper-based questionnaire responses were first digitized and manually entered into a Microsoft Excel spreadsheet for analysis. The Likert-scale responses were summarized by grouping related response categories to simplify interpretation and amplify clarity of outcomes (ie, for each Likert-scale item, responses were categorized into 3 groups: positive [eg, “Trustworthy” and “Very Trustworthy”], negative [eg, “Untrustworthy” and “Very Untrustworthy”], and neutral or uncertain midscale responses]). After categorizing responses into these groups, the frequency and proportion of participants falling into each category (positive, negative, neutral, or uncertain) were calculated. Demographic information was analyzed by summarizing frequencies and proportions in each demographic category. The summarized data were presented using descriptive statistics, such as percentages or frequencies, to clearly illustrate the distribution of responses and identify meaningful patterns or changes attributable to the intervention.

#### Qualitative Data

A broad, open-ended questionnaire question (“Do you have any thoughts?”) was included to gather richer, detailed insights beyond what the quantitative Likert-scale responses could capture. These written responses were digitized from paper questionnaire sheets and entered into a Microsoft Excel database for analysis. A thematic textual analysis was conducted following a structured inductive approach, which involved systematically coding the qualitative data to identify recurring patterns and significant themes. The initial codes were generated by highlighting salient words, phrases, or sentences representing key ideas and experiences expressed by participants. These initial codes were then grouped and categorized into broader themes, such as perceived advantages of robot usage, concerns and apprehensions, suggestions for improvements, emotional reactions, and reflections on robot-human interactions.

### Ethical Considerations

The study was reviewed and approved by the Research Ethics Committee of the National Institute for Health Development in Estonia (#8.3/13‐24 from May 29, 2024). All participant interactions with TEMI v3 were anonymous, and no patient or visitor data were recorded. Participants were informed about the purpose of the study, their voluntary participation, and the nonrecording of identifiable data. Participants received no compensation for their participation. Informed consent was obtained from all participants and clear instructions were given regarding robot interactions, particularly in the patrol scenario, to ensure privacy and security protocols were followed.

## Results

### Response Overview

**Figure 1.**
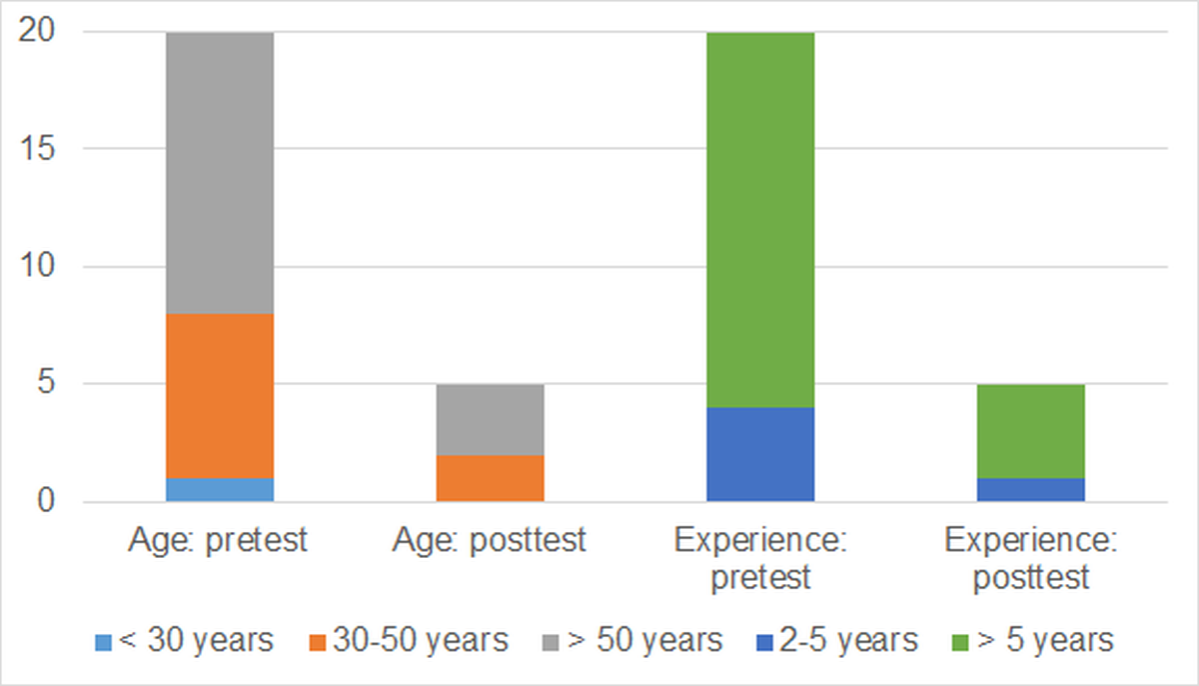
The age and work experience of the participants who filled in the pre- and posttest questionnaires.

20 responses were received to the initial pretest questionnaire, which provided a broad overview of the attitudes and demographic information of participating health care workers. As the unit was relatively small, with administrative personnel having previous long-term experience as ordinary older adult health care employees, we did not collect separate information about the employees’ current positions. The posttest questionnaire was completed only by 5 participants, representing a significant attrition rate of 75% (n=15). The possible reasons are discussed under the “Limitations” section. The professional experience duration of participants, together with their age, is shown in Figure 1.

As demonstrated in Figure 1, the majority of participants had more than 5 years of experience in the health care sector and were older than 50 years. In the posttest group, similar tendencies were seen.

### Quantitative Results

#### Pretest Results

**Figure 2.**
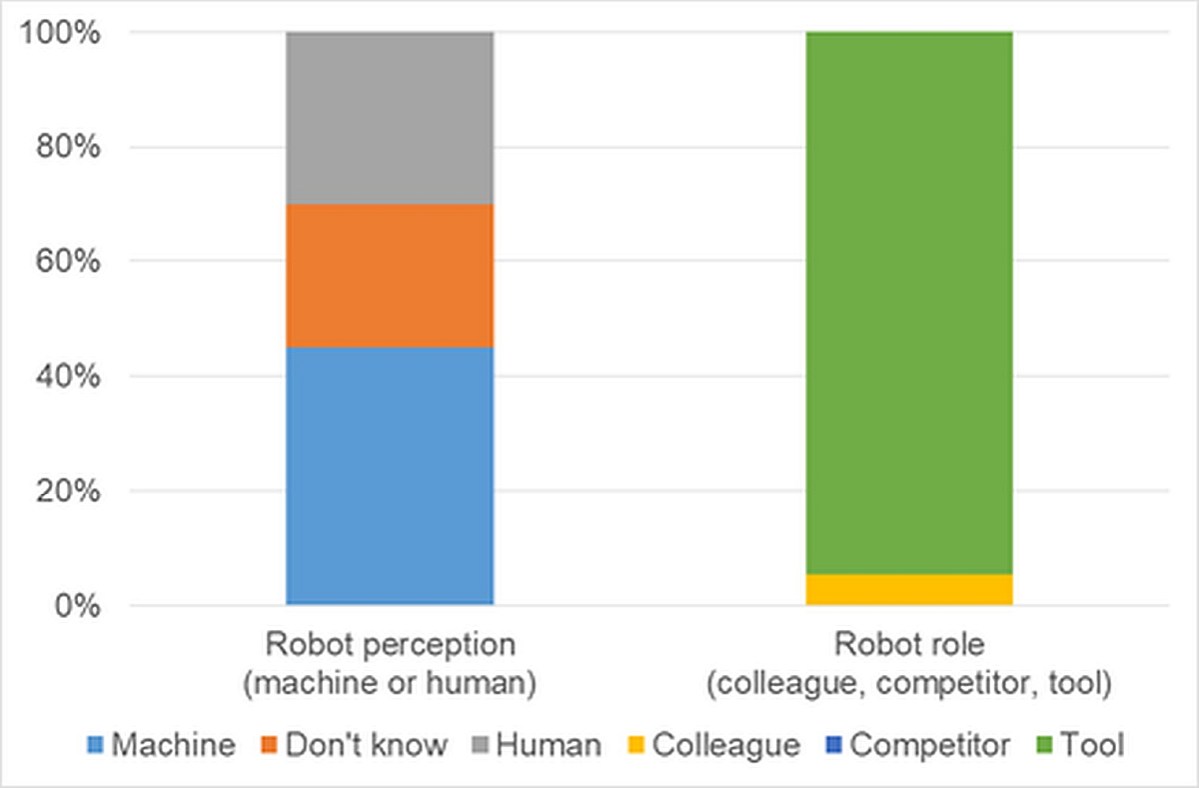
Percentage distribution of responses for robot perception and role in the workplace.

Of the pretest respondents, 85% (n=17) reported no significant prior experience with robots in their work, 10% (n=2) had some experience, and 5% (n=1) used robots frequently. When asked if they saw robots as colleagues or competitors, 5% (n=1) saw them as colleagues, 95% (n=19) saw them as tools, and none saw robots as competitors (Figure 2, right). Almost half of the participants (45%, n=9) considered robots to be machines, 30% (n=6) perceived them similar to humans, while one quarter of participants (n=5) were unsure (Figure 2, left).

**Figure 3.**
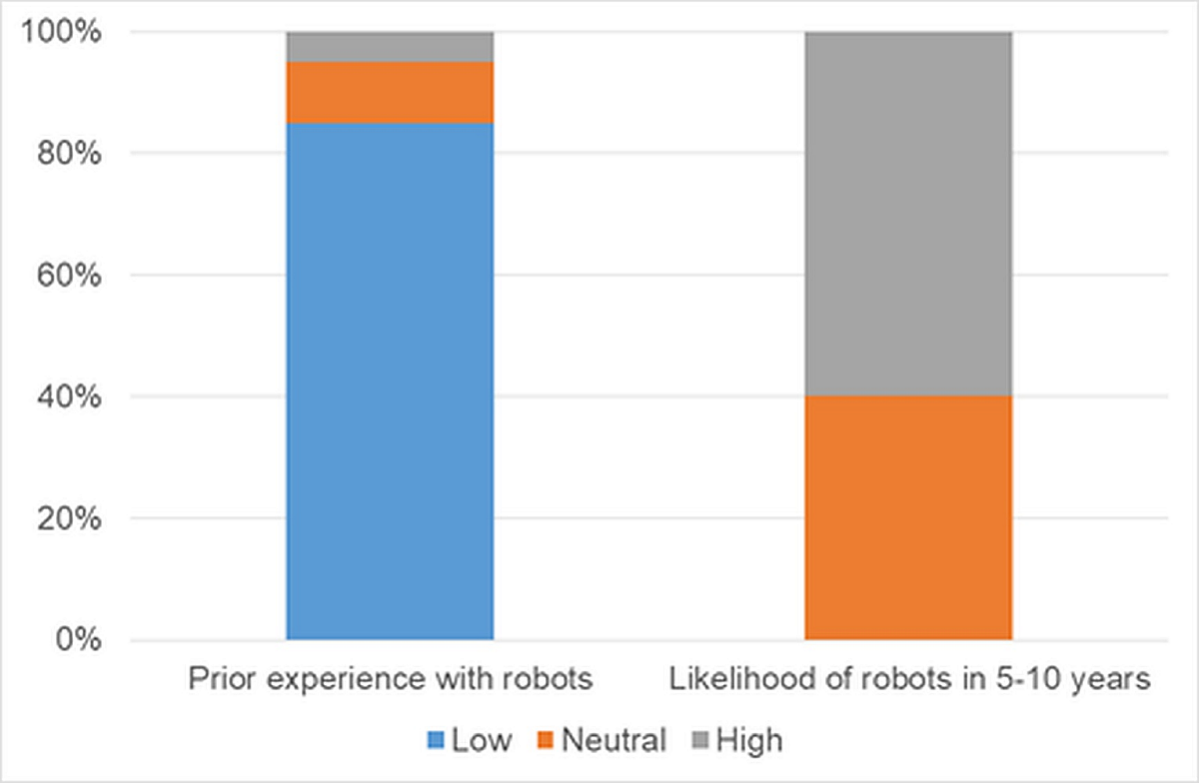
Percentage distribution of responses for previous experience with robots and likelihood of robots in the next 5‐10 years.

Participants also shared their previous experience with SARs; 85% (n=17) had low experience with them, 10% (n=2) had insignificant experience, and 5% (n=1) had considerable experience with SARs (Figure 3, left). Regarding the potential for robots to become mainstream in their field in the next 5‐10 years, 60% (n=12) thought it was realistic, while 40% (n=8) were unsure (Figure 3, right).

**Figure 4.**
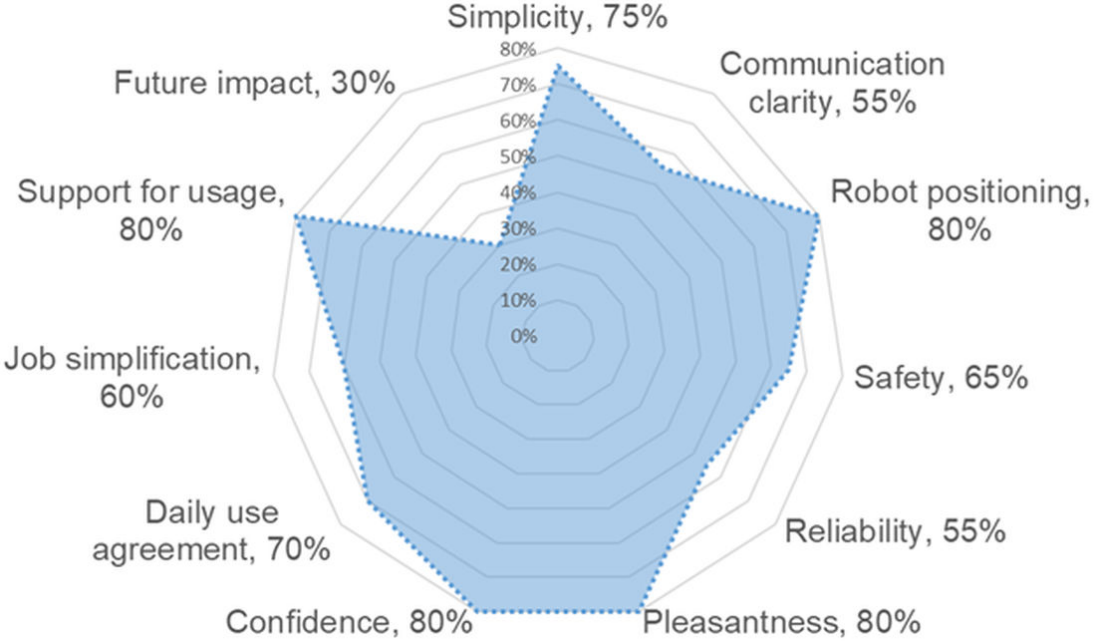
Percentage distribution of high ratings (5-7) across robot collaboration attributes, pretest.

Participants then rated specific aspects of working with robots (see Figure 4). When it comes to collaboration simplicity, 75% (n=15) found it easy, 20% (n=4) were unsure, and 5% (n=1) found it a challenge. Communication clarity with the robot was rated good by 55% (n=11), while 30% (n=6) were not sure and 15% (n=3) rated it bad. The height of the robot was considered suitable by 80% (n=16) of respondents, 20% (n=4) were undecided.

In terms of safety, 65% (n=13) of respondents believed that robots are safe, 20% (n=4) were not sure, and 15% (n=3) felt that robots were not safe. 55% (n=11) of participants considered robots trustworthy, while 35% (n=7) were unsure and 10% (n=2) considered them untrustworthy. A significant 80% (n=16) of respondents found robots to be *pleasant*, although 20% (n=4) were undecided. Regarding *c*onfidence in working with robots, 80% (n=16) felt confident, 5% (n=1) were unsure, and 15% (n=3) were uncertain.

When considering the integration of robots into daily work, 70% (n=14) were ready, 15% (n=3) were not, and 15% (n=3) were unsure. Regarding the potential of robots to make their jobs easier, 60% (n=12) agreed, 20% (n=4) disagreed, and 20% (n=4) were unsure. In addition, 80% (n=16) were ready to promote the use of robots in their workplace, while 20% (n=4) were unsure. Finally, when asked about the impact of robots on the future of their work, 30% (n=6) thought robots would have an impact, while 35% (n=7) thought that robots would not have any impact and another 35% (n=7) were unsure.

#### Posttest Results

Among the posttest respondents, 100% (n=5) reported some experience with robots. Regarding the potential for robots to become mainstream in their field within the next 5‐10 years, 60% (n=3) found it realistic, with the remainder undecided. In terms of perception, all participants identified the TEMI V3 robot as a machine, with no participants considering it human-like. When asked whether robots would be viewed as colleagues, tools, or competitors, 100% (n=5) considered them as tools.

For specific aspects of working with robots, 80% (n=4) of participants felt that collaboration with robots was straightforward, while 20% (n=1) were uncertain. Communication clarity was rated positively by 80% (n=4) of respondents, with 20% (n=1) uncertain. The robot’s height was deemed appropriate by 80% (n=4), with 20% (n=1) undecided.

Regarding safety, 100% (n=5) of respondents felt that robots were safe. Robots were deemed trustworthy by 60% (n=3), while 40% (n=2) were uncertain. Furthermore, all respondents found robots pleasant. For self-confidence in working with robots, 80% (n=4) felt assured, and 20% (n=1) were uncertain.

On the integration of robots into daily tasks, 80% (n=4) were willing to incorporate them into their work, and 20% (n=1) were uncertain. Regarding robots’ potential to facilitate work, 60% (n=3) disagreed, with 20% (n=1) uncertain and 20% (n=1) agreed. In addition, 80% (n=4) were prepared to advocate for robot use in the workplace, while 20% (n=1) were unsure. Finally, regarding the impact of robots on future work, 40% (n=2) believed robots would have an impact, 40% (n=2) did not think so, and 20% (n=1) were undecided.

#### Comparison of Pre- and Posttest Results

In both the pretest and posttest groups, participants expected robots to become mainstream in the clinical care of older adults within the next 5‐10 years. However, some changes in robot perception and comfort were observed between the results of the 2 tests, suggesting that there was an increase in positive perceptions and confidence in collaborating with robots, particularly regarding safety, reliability, and the potential role of robots in the workplace.

In the pretest, 45% (n=9) of respondents perceived TEMI V3 as a machine, 30% (n=6) found it somewhat human, and 25% (n=5) were not sure. In the posttest, all participants (n=5) consistently identified the TEMI v3 robot as a machine, indicating a shift towards viewing robots as nonhuman tools. Comfort and confidence in working with robots also increased in the posttest. Perceptions of safety improved significantly, with 100% (n=5) of posttest respondents considering the robots to be safe, compared to 65% (n=13) in the pretest. Reliability ratings similarly increased, with all posttest respondents (n=5) finding robots enjoyable to work with, while 20% (n=4) in the pretest were undecided.

However, it seems that after having an actual experience with SARs, the participants’ belief in the robots’ ability to simplify their work decreased. Despite this, posttest respondents showed a stronger willingness to integrate robots into their daily work (80%, n=4 ready compared with 70%, n=14 pretest) and a greater willingness to promote the use of robots in the workplace. This openness extended to confidence about the impact of robots on future work, with a slight increase in the number of people expecting a positive impact.

### Qualitative Results

**Figure 5.**
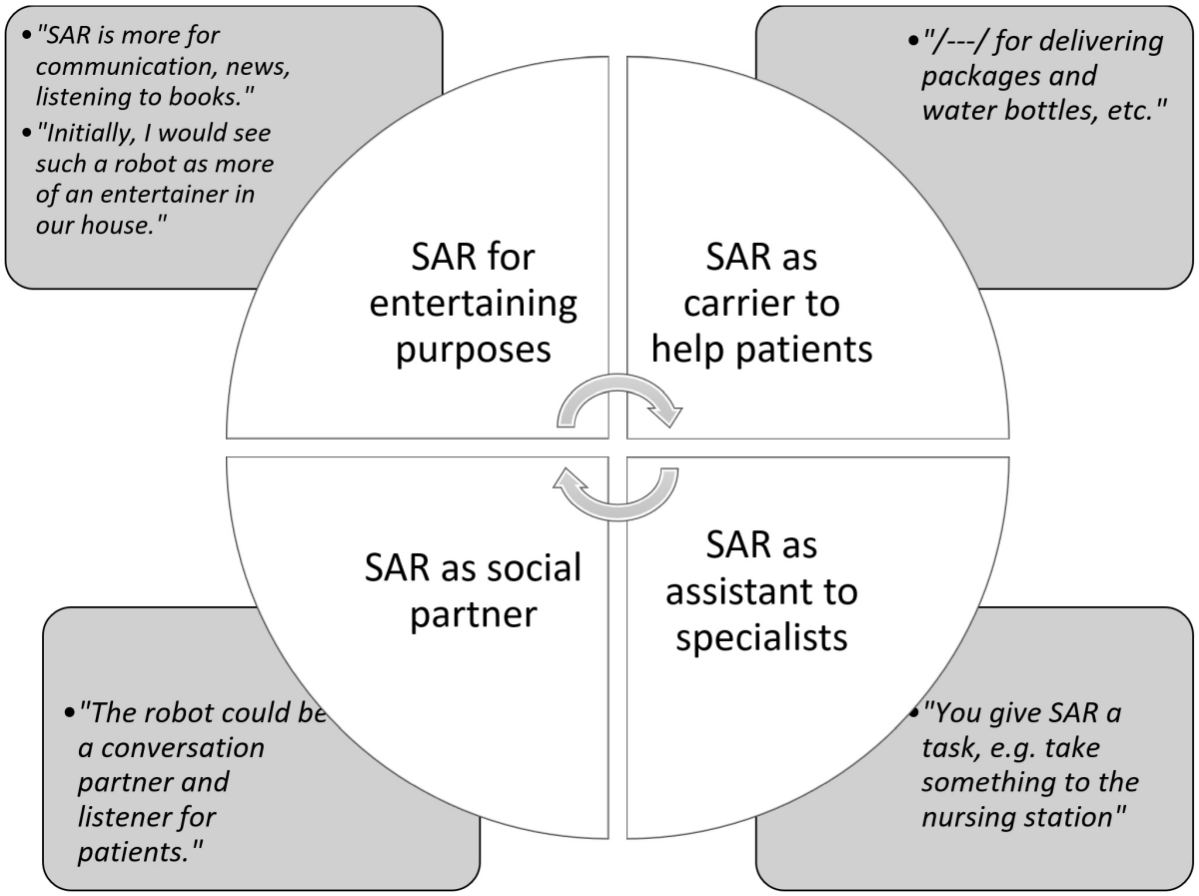
Potential roles for socially assistive robots (presented with illustrative quotes). SAR: socially assistive robots.

An open-ended questionnaire question (“Do you have any thoughts?”) provided detailed insights in addition to the quantified data from the Likert-scale responses. Digitized responses underwent thematic textual analysis, key phrases were highlighted as initial codes and grouped into broader themes to identify recurring patterns. The main themes of qualitative analysis are seen in Figure 5.

As seen from Figure 5, participants proposed several functional and emotional roles for the TEMI robot in a health care facility. Many envisioned TEMI as a mobile assistant that could be summoned via a Wi-Fi-connected station, useful for tasks such as delivering items to the nurse’s station, reducing the need for staff to carry them manually. TEMI was also seen as a tool to improve the patient experience, provide entertainment, deliver items, and be a conversational partner. Respondents felt that TEMI could lift patients’ moods by providing social interaction, news, and audiobook playback.

## Discussion

### Principal Findings

This study explored the potential benefits and challenges of integrating SARs, such as TEMI v3, into nursing care facilities. SARs provide value in supporting health care workers by performing noncore tasks such as guidance, delivery, and patrolling, thereby allowing staff to focus on complex interpersonal care tasks. However, the implementation of SARs in health care also presents a variety of emotional, professional, and technical challenges that need to be addressed through thoughtful strategies.

One of the most important findings is the importance of balancing technological efficiency with the emotional needs of older adult patients. The “human touch” is crucial in aged care, where personal interaction promotes emotional support and social inclusion [27]. Consistent with previous research, our results suggest that health care professionals were concerned about SARs replacing human roles that require empathy and warmth and perceived that SARs lacked the human-like intuitive and comforting qualities that patients value [21]. Similar findings were observed by Turja et al [23,31], who reported that SARs may have problems achieving the warmth needed in their caregiving roles, which may lead to resistance from patients and caregivers. By positioning SARs in complementary roles, such as logistical support rather than personal care, we sought to preserve the human aspects of care while benefiting from the robot’s hands-on capabilities. This “complementarity model” aligns with the view of Sharkey and Sharkey [27], who advocate SARs as an aid rather than a substitute in situations that require emotional connection. In addition, transparent communication and role clarification, emphasizing that SARs are designed to support, not replace, staff, are essential to addressing these issues.

Possible generational differences in SAR acceptance must be considered. Czaja and Lee [32] found that younger people are generally more adaptable to new technologies. These findings suggest that younger health care workers could be more susceptible to SARs, implying that deployment strategies need to include tailored training and engagement. These findings highlight the importance of addressing job security fears as part of a holistic approach to SAR integration. Research also shows that older adults often experience technophobia, which is defined as fear or anxiety of technology [24]. Consistent with our findings, Barnard et al [25] and Yusif et al [33] suggest that older health workers may have concerns about adapting their routines to SARs. To mitigate this barrier, structured, scenario-based training sessions should be designed to introduce staff to SARs in a supportive, low-stakes environment, building trust and reducing resistance. Papadopoulos et al [11], emphasize that participatory training significantly improves technology adoption by empowering users to adopt new tools. Our study’s training approach is consistent with these findings, as it provided staff with hands-on interactions that increased their comfort and understanding, ultimately promoting a more inclusive environment for SAR adoption.

The issue of effective training of SARs needs to be approached systematically, because even when fears about new technology are addressed and workers have had time to familiarize themselves with SARs, integrating these robots into the clinic’s everyday life remains a challenge. Interestingly, the number of people who thought SAR did not make their work easier, already high at the start of the study, increased after 2 weeks of exposure, while the number of people who were ready to integrate robots to their daily routines also stayed high. This increase suggests that 2 key factors may be necessary for the successful implementation of SARs in aged care settings. First, a high level of customization is required to ensure that SARs meet the specific demands of aged care. For example, in our experiment, the software design lacked an iterative development approach that would have allowed user feedback to shape and refine the robot’s features and behavior. Second, it seems important to equip health care professionals with established methods and routines tailored to meaningfully incorporate SARs into their workflows. This dual approach—favoring iterative, user-driven customization with the provision of well-defined routines—may play an important role in increasing the perceived utility and acceptance of SARs in daily health care practices.

Although participants found SARs relatively easy to use, this does not mean that these robots inherently facilitate human tasks unless their implementation is carefully planned. For SARs to be truly effective, health care professionals need to be confident that robots can be trusted to provide real support, and it is up to senior stakeholders, such as management, to clearly demonstrate how SARs can positively impact staff’s daily routines. Furthermore, it is possible that SARs may not significantly reduce the workload of health care professionals, but rather provide greater value by improving the quality of life and meaningful experiences for patients. It is important to understand whether SARs can fulfill this role effectively, and future research should explore this potential by assessing the long-term impact of SARs on both patient well-being and health care provider job satisfaction.

### Limitations

Some limitations of the study must be acknowledged. First, SARs were tested in one nursing clinic in a relatively short period of time, so the findings cannot be generalized to all Estonian health care workers. Second, the study site was located in the capital of Estonia, and the study does not provide an overview of possible differences in perceptions in rural or peripheral areas, which could be important in terms of general trust in the use and ability to use technology in older adults care settings. Third, due to piloting in a single care clinic, we also had a limited sample of 45 participants with a participation rate of <50% in the pretest phase and even lower in the posttest period. This decline is likely due to a combination of the following factors: (1) loss of interest in the technology—some participants may have lost interest in SARs after initial engagement, perceiving limited personal benefits or finding the technology less impactful than expected; and (2) focus on primary responsibilities—it is likely that, for reasons discussed in the introduction, participants found it difficult to allocate extra time to nonessential tasks such as the SAR study. In addition, the implementation scenarios for Temi v3 robot in this study were specifically designed to act on an auxiliary basis, without impact on the day-to-day care processes (ie, the robot did not participate independently directly in treatment nor tendance, making it more difficult for the employees to grasp the beneficial impact of using the robot on their tasks). The study did not include nursing clinic patients and focused on the perspective of health care professionals, so it is not clear whether and how professionals’ perceptions would change if patients liked or disliked SARs, eg, whether acceptance would be higher or fears and emotions lower if patients perceived SAR as useful and acceptable. Despite all these limitations, we believe that this study provides valuable information on the potential of implementing SARs in nursing clinical care, outlining areas that need to be considered in future research and implemented in daily practice.

### Implications for Future Research and Practice

The results of the study confirm the need for a step-by-step and inclusive approach to the integration of SARs that considers the emotional dimensions of older adults’ care, concerns about job displacement, and varying levels of technological readiness. Future research should explore strategies to maintain engagement among all age groups, particularly among older health professionals who may find SARs distracting or irrelevant. Longitudinal studies can provide deeper insight into how SARs affect patient and staff experiences over time, allowing researchers to track changes in perceptions as SARs become more common in health care settings. In addition, continued exploration of SARs in different roles, such as logistics and routine support, is vital to identify where SARs add the most value without compromising the essential “human touch” of aged care.

## Abbreviations

CARESSES: Culture-Aware Robots and Environmental Sensor Systems for Elderly Support
HRI: human-robot interaction
SAR: Socially Assistive Robot
TAM: Technology Acceptance Model
TRI: Technology Readiness Index
UTAUT: Unified Theory of Technology Acceptance and Use
